# Subthalamic control of impulsive actions: insights from deep brain stimulation in Parkinson’s disease

**DOI:** 10.1093/brain/awae184

**Published:** 2024-06-13

**Authors:** Damian M Herz, Michael J Frank, Huiling Tan, Sergiu Groppa

**Affiliations:** Movement Disorders and Neurostimulation, Department of Neurology, Focus Program Translational Neuroscience (FTN), University Medical Center of the Johannes Gutenberg-University Mainz, 55131 Mainz, Germany; Department of Cognitive, Linguistic and Psychological Sciences, Carney Institute for Brain Science, Brown University, Providence, RI 02903, USA; MRC Brain Network Dynamics Unit at the University of Oxford, Nuffield Department of Clinical Neurosciences, University of Oxford, OX1 3TH Oxford, UK; Movement Disorders and Neurostimulation, Department of Neurology, Focus Program Translational Neuroscience (FTN), University Medical Center of the Johannes Gutenberg-University Mainz, 55131 Mainz, Germany

**Keywords:** subthalamic nucleus, basal ganglia, local field potentials, decision-making, cognitive control, impulse control disorders

## Abstract

Control of actions allows adaptive, goal-directed behaviour. The basal ganglia, including the subthalamic nucleus, are thought to play a central role in dynamically controlling actions through recurrent negative feedback loops with the cerebral cortex. Here, we summarize recent translational studies that used deep brain stimulation to record neural activity from and apply electrical stimulation to the subthalamic nucleus in people with Parkinson’s disease.

These studies have elucidated spatial, spectral and temporal features of the neural mechanisms underlying the controlled delay of actions in cortico-subthalamic networks and demonstrated their causal effects on behaviour in distinct processing windows. While these mechanisms have been conceptualized as control signals for suppressing impulsive response tendencies in conflict tasks and as decision threshold adjustments in value-based and perceptual decisions, we propose a common framework linking decision-making, cognition and movement. Within this framework, subthalamic deep brain stimulation can lead to suboptimal choices by reducing the time that patients take for deliberation before committing to an action. However, clinical studies have consistently shown that the occurrence of impulse control disorders is reduced, not increased, after subthalamic deep brain stimulation surgery. This apparent contradiction can be reconciled when recognizing the multifaceted nature of impulsivity, its underlying mechanisms and modulation by treatment. While subthalamic deep brain stimulation renders patients susceptible to making decisions without proper forethought, this can be disentangled from effects related to dopamine comprising sensitivity to benefits versus costs, reward delay aversion and learning from outcomes.

Alterations in these dopamine-mediated mechanisms are thought to underlie the development of impulse control disorders and can be relatively spared with reduced dopaminergic medication after subthalamic deep brain stimulation. Together, results from studies using deep brain stimulation as an experimental tool have improved our understanding of action control in the human brain and have important implications for treatment of patients with neurological disorders.

## Teaser

Herz *et al.* review recent studies that have used deep brain stimulation electrodes to record neural activity from, or apply electrical stimulation to, the subthalamic nucleus in people with Parkinson’s disease. The results provide insights into action control in the human brain, with implications for the treatment of neurological disorders.

## Introduction

**Figure 1:**
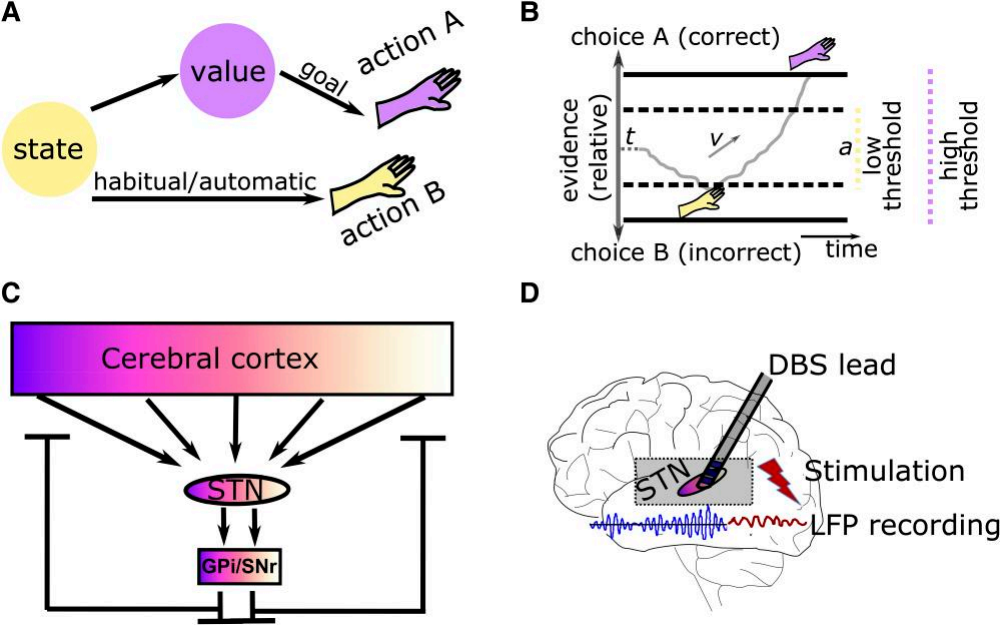
**Conceptual framework and experimental model.** (**A**) Goal-directed behaviour (purple) relies on assigning expected outcomes to actions, which can then be chosen based on optimization of subjective value. In contrast, in habitual or automatic behaviour (yellow), a certain state or stimulus directly maps to an action. This direct mapping is thought to be faster and less computationally costly compared to goal-directed behaviour. (**B**) In sequential sampling models, evidence (grey) for one option versus another is accumulated over time until it reaches the decision threshold *a* (horizontal black line). With low thresholds (dashed horizontal lines), the option is chosen quicker but is more likely to be incorrect (here choice B in yellow), while higher thresholds (continuous horizontal lines) lead to more accurate but slower choices (here choice A in purple). Parameters *t* and *v* refer to, respectively, non-decision time (e.g. early sensory processing) and drift rate (rate of evidence accumulation). (**C**) A simplified illustration of cortical-subthalamic nucleus (STN) connectivity shows that cortical organization (purple to white gradient) is also reflected in STN and basal ganglia output structures [internal pallidum (GPi) and substantia nigra pars reticulata (SNr)] but with considerable overlap. There is a tremendous dimensionality reduction from cortex to STN, which has a net neural inhibitory effect on cortex and descending output. (**D**) Illustration of a deep brain stimulation (DBS) lead, which can be used for recording activity from and apply stimulation to the STN. LFP = local field potential.

Movements enable us to interact with our environment. Rather than simply predicting whatever happens next, we can adapt our behaviour to reach our goals and avoid harm.^1^ But how do we make sure that our actions are in line with our goals? Cognitive control refers to a set of functions in the brain that monitor our environment and behaviour, and implement changes of our actions when warranted.^2,3^ One of the main objectives of cognitive control is to enable goal-directed behaviour. In contrast to automatic or habitual behaviour, in which a certain stimulus or state maps directly to an action, goal-directed behaviour, reflects an effortful process allowing more flexibility, e.g. when the value of an outcome or action-outcome contingencies have changed.^4-6^ Thus, in some circumstances, we need to suppress automatic, fast responses to select our actions based on slower cognitive processes (Fig. 1A). This is thought to be implemented by cognitive control facilitating the allocation of resources such as attention and working memory to our actions when necessary.^3,7,8^

A similar process determining whether we should act fast or slow is studied in the field of decision-making. Decision-making is concerned with the computations involved in comparing two or more options, e.g. regarding their expected value in value-based decision-making or sensory properties in perceptual decision-making.^9-11^ Weighing the time we spend on deliberation (speed) versus the likelihood of arriving at the correct choice (accuracy) is central for optimizing decisions.^12,13^ Mathematically, this can be conceptualized as an increased level of evidence that needs to be accumulated before an action is selected, often referred to as ‘decision threshold’ in sequential sampling models.^14,15^ The decision threshold can be elevated when faced with conflicting information, increased task difficulty or after a mistake^16-20^ (Fig. 1B). Such dynamic adjustments of decision thresholds are similar to cognitive control in that they allow people to switch to more cautious ways of selecting their actions when necessary, while routinely relying on simpler decisions requiring less evidence or even selecting actions without assigning any explicit value computations.^5,21-23^

Thus, the ability to control fast but putatively suboptimal actions to allow slower, more accurate choices is a crucial process for optimizing behaviour. Traditionally, cognitive control and decision-making have been studied in separate fields, the former focusing mainly on peoples’ abilities to override automatic responses for allowing deliberate choices, and the latter primarily studying the benefits and costs of different options. However, there is mounting evidence for a large overlap between the involved mechanisms.^24^ In particular, both critically rely on optimizing the time we take before committing to a choice, which can be parsimoniously conceptualized as the controlled delay of an action. How might this process be implemented in the brain?

The basal ganglia (BG) consist of a network of nuclei seated deep in the brain common to all vertebrates.^25^ The BG receive high dimensional somatotopic afferents from cortical neurons (primarily layer 5) and send low dimensional feedback to the cortex in a recurrent negative feedback architecture, which also exists at the subcortical level.^26-29^ Cortico-BG loops originating from different cortical areas are organized in parallel but overlapping circuits showing a cortical rostro-caudal gradient,^30,31^ illustrated in Fig. 1C. The glutamatergic subthalamic nucleus (STN) receives input from D2-expressing GABAergic neurons of the striatum relayed via GABAergic prototypic neurons of the external pallidum in the indirect pathway and monosynaptic glutamatergic input from cortex in the hyperdirect pathway, as well as afferents from multiple subcortical areas.^32,33^ Both the indirect and hyperdirect pathways exert a net inhibitory effect on their output structures.^25,27^ Given the immense dimensionality reduction (roughly a 1000-fold reduction in the number of neurons from striatal BG input to BG output^26,34^), the very fast monosynaptic connection between cortex and STN, as well as the net inhibitory effects of subthalamic activation, the STN has been proposed to mediate fast and broad inhibition of neural activity resulting in a pause of behaviour.^13,16,35-38^

At the cortical level, several areas of the prefrontal cortex, in particular (pre)-supplementary motor area, dorsal anterior cingulate cortex and right inferior frontal gyrus are thought to be neural hubs evaluating information such as the presence of conflict, unexpected events or negative feedback to recruit the necessary control mechanisms.^3,6,16,39,40^ Importantly, the prefrontal cortex has direct connections to the STN through the hyperdirect pathway innervating the STN just ventro-medially to and overlapping with subthalamic regions mainly connected to motor cortical areas.^31,41-43^ Thus, the architecture of cortico-STN networks seems well suited to implement the controlled pause or delay of an action, which has been supported by evidence in non-human primates.^44,45^ This allows the avoidance of fast but putatively suboptimal actions for slower, more accurate choices.^46,47^ However, this hypothesis is difficult to test in humans, since the deep-seated localization and small size of the STN make it notoriously difficult to record activity from the STN or interfere with its function non-invasively. This challenge can be overcome by studying patients with Parkinson’s disease (PD) who are treated with STN deep brain stimulation (DBS) for clinical purposes.

PD is a frequent, disabling neurological disorder characterized by progressive degeneration of multiple neuromodulatory systems, in particular dopaminergic cells in the midbrain.^48^ PD can lead to various neuropsychiatric symptoms.^49^ It is defined clinically by the hallmark presence of movement slowness (bradykinesia), as well as other motor symptoms such as shaking (tremor) and increased muscle stiffness (rigidity).^49,50^ The mainstay treatment of PD is dopamine replacement. In case of complications to medical treatment, e.g. movement fluctuations and involuntary movements (dyskinesias), or insufficient effects on tremor, symptoms can be alleviated by DBS.^51^ During DBS surgery, electrodes are implanted in subcortical structures, in PD most commonly the STN, and connected to a subcutaneous implantable pulse generator (IPG). For symptom alleviation, stimulation is given continuously at high frequencies (>100 Hz) at intensities of several mA, depending on the target structure and the clinical response to DBS. The mechanisms of DBS are multifaceted at different temporal and spatial scales.^51-54^ Functionally, the effects of STN-DBS are in line with a reduced excitatory influence of cortical and increased inhibitory influence of subcortical areas on the STN, which has been described as a functional or informational lesion.^53,55^

Besides its important role in clinical therapy, DBS can also be used as an experimental tool to better understand how the brain mediates behaviour.^56-59^ In some DBS centres, surgery is performed in two stages, where electrodes are implanted and externalized in the first stage and only connected to the IPG (i.e. internalized) in the second stage ∼1 week after the first operation.^51^ This allows the recording of local field potentials (LFPs) from the STN or application of electrical stimulation through the externalized extension cables before internalization of the device (Fig. 1D). Novel DBS devices allow simultaneous stimulation and recordings of STN LFPs in patients with implanted IPGs without the need for electrode externalizations.^51^

Invasive STN recordings have revealed pathological activity patterns in PD underlying movement slowness^60,61^ and therapy-related involuntary dyskinesia movement.^62,63^ However, they can also be used to infer physiological STN functions in controlling behaviour. Of note, when inferring physiological functions of the STN, it is important to consider that the studied participants are not healthy but suffer from a neurological disorder. To address this, most DBS studies discussed here tested PD patients ON their dopaminergic medication, which is thought to normalize neural activity and behaviour as much as possible. However, since dopamine medication can in itself lead to abnormal behaviour,^64,65^ it is helpful to include a healthy control group for behavioural testing and to conduct complementary neurophysiological studies in healthy non-human animals or humans suffering from other disorders, which can be treated by DBS surgery, such as obsessive compulsive disorders. Thus, taking these considerations into account, DBS gives the unique opportunity to record neurophysiological activity in humans from the STN that cannot easily be recorded non-invasively and to modify brain activity and behaviour through temporally and spatially focused electrical STN stimulation. In this review, we will first consider previous studies recording neural activity from the STN during tasks probing cognitive control and decision-making, before summarizing the behavioural effects of STN DBS and finally discussing clinical implications.

## Recording neural activity through deep brain stimulation electrodes

**Figure 2:**
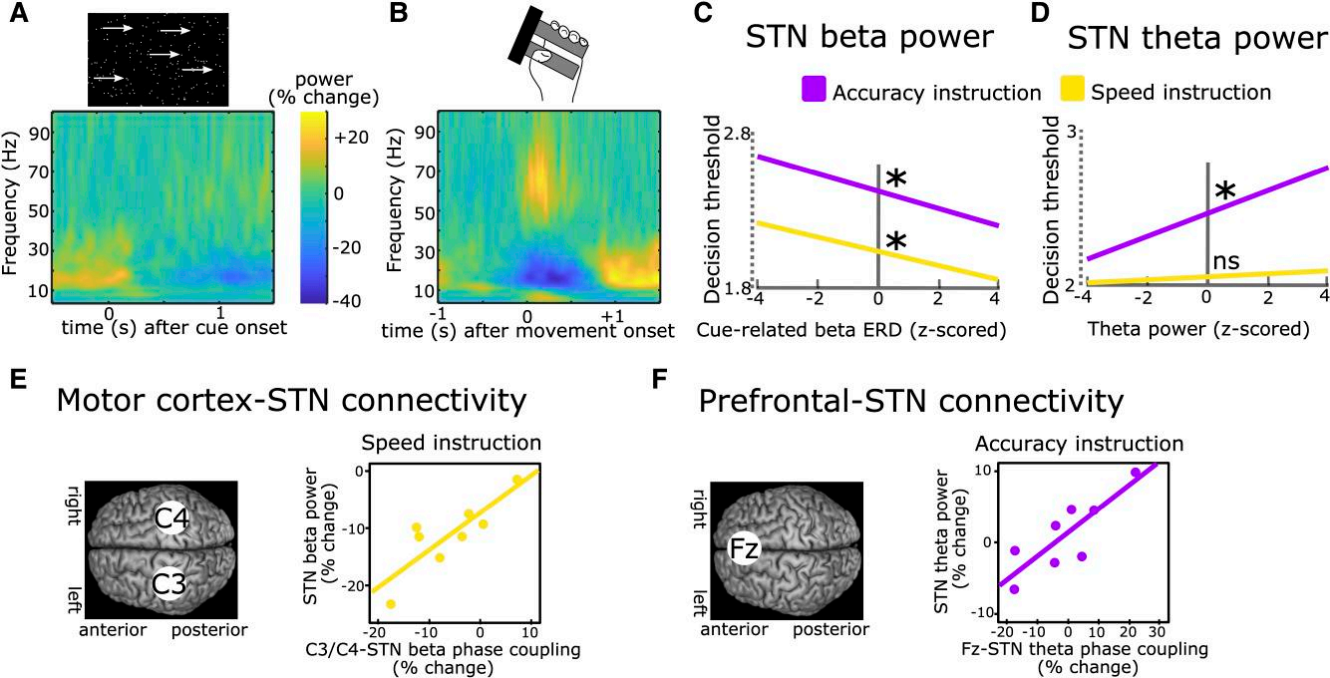
**Recordings of subthalamic activity.** (**A**) Time-frequency spectrum of subthalamic nucleus (STN) recordings (group-level) aligned to the onset of a moving dots-cue. (**B**) Same as in **A** but aligned to onset of movement (force grip). (**C**) Stronger cue-induced reductions in STN beta power are predictive of lower decision thresholds irrespective of instructions. (**D**) Increases in STN theta power during deliberation are predictive of higher decision thresholds after Accuracy (purple) but not Speed instructions (yellow). (**E**) Phase coupling between electrode C3/C4 (covering motor cortex) and STN in the beta band correlates with STN beta power during Speed (yellow) but not Accuracy instructions (not shown). (**F**) Phase coupling between electrode Fz (covering medial prefrontal cortex) and STN in the theta band correlates with STN theta power during Accuracy (purple) but not Speed instructions (not shown). **A** and **B** are based on Herz *et al*.^73^**C**–**F** are based on Herz *et al*.^69^

Invasive STN recordings during behavioural tasks using externalized DBS leads have been conducted for over 20 years.^60^ The strongest task-modulated LFP signal is the beta band (∼13–30 Hz), which, similar to motor cortical beta power, is reduced just prior to and during movement as well as after salient cues and increases after movement termination. This is often termed beta event-related desynchronization (ERD) and synchronization (ERS), respectively. Furthermore, increases in low frequency activity around the theta band (∼2–8 Hz; the theta band is usually defined as 4–8 Hz in EEG but can extend to even lower frequencies at ∼2 Hz in the STN) are often seen after cues and around movement, similar to frontal cortical theta power. Finally, there are strong increases in broad-band gamma activity (∼50–90 Hz) at movement onset (for an example see Fig. 2A and B). These general task-related changes in the LFP are highly reproducible and have been reported throughout a range of different tasks.^66-72^ Task-related changes in these frequency bands can be used as a correlative measure to examine whether modulations of spectral STN activity correspond to a given (observable or latent) process of interest.

There is mounting evidence that dynamic modulation of STN theta and beta activity are reflective of processes related to suppressing and delaying actions. These modulations can be observed in studies of cognitive control and decision-making and can be related to behavioural adjustments at the single trial level. Yet, there are important distinctions between subthalamic control of actions in the theta and beta band, particularly regarding their modulation by task context, which we will discuss in more detail below.

In an early study,^74^ patients had to push a button with their right or left hand after an imperative cue. The direction (right versus left) was indicated by a preceding warning cue. In 20% of trials, the imperative cue was replaced by another cue signalling that participants had to refrain from executing the movement. In all conditions, beta power was reduced after each cue, but this decrease was terminated earlier in trials requiring movement suppression leading to a relative increase of beta power. Furthermore, inter-individual differences in the latency of the cue-related beta ERD were correlated with differences in mean reaction times. The relative increase in STN beta power in trials with movement inhibition versus execution was confirmed in several other studies.^75-80^ These findings were mainly interpreted as STN beta ERD allowing movement execution, which could be aborted by a relative increase in STN beta power. However, the relative difference between conditions might have simply reflected the absence of a movement-related beta decrease in conditions with movement inhibition since STN beta power is strongly reduced during movements (as shown in Fig. 2B). Furthermore, since no action is executed during movement suppression, it is difficult to relate modulations of STN beta power to trial-by-trial changes in behaviour. One way to address this is to induce a longer deliberation period between imperative cue and movement (and thus between the cue-related and movement-related beta ERD) and to use computational modelling to reveal latent cognitive mechanisms. In a recent study, participants performed a moving-dots task^81^ (Box 1) in which they had to decide whether a cloud of dots appeared to move to the left or to the right and then press a button with the corresponding hand.^69^ When patients were instructed to weigh accuracy over speed, they had increased reaction times and committed fewer fast errors. Computational modelling using a drift diffusion model^15^ revealed that this could be explained by an increase in the decision threshold. STN beta power showed a marked reduction after the moving dots cue, which was steeper in the Speed condition favouring fast over accurate choices. The steeper beta was reduced on a trial-by-trial basis, the faster were reaction times and the lower were decision thresholds (Fig. 2C). Importantly, responses occurred much later (∼1 s) on average than the duration of the cue-induced beta ERD and the relationship between STN beta power and, respectively, reaction times and decision thresholds was preserved when excluding any trials where responses fell into the beta ERD window. Thus, the relatively higher beta power in trials with increased decision thresholds could not simply be explained by movements already occurring in this time period when decision thresholds were low. This finding has since been replicated twice.^17,73^ Furthermore, it was demonstrated that decision thresholds were specifically reflected by modulations of the cue-induced beta ERD, while the later occurring movement-related beta ERD correlated with movement, not decision, speed.^73^

Box 1 **Commonly used experimental tasks**

**Eriksen-Flanker task:** The correct response is indicated by a central arrow, which is flanked by distracting arrows. In incongruent trials the central arrow points to a different direction compared to the flanking arrows. Different versions of the task exist using letters or numbers.

**Moving dots task:** A cloud which consists of many white dots is shown on a screen. While some dots move coherently to one direction (e.g. 8% of all dots in more difficult trials or 50% of dots in easier trials) the remaining dots move randomly. Participants have to decide whether the cloud appears to move to the left or right and indicate this choice usually by a left- versus right-hand movement.

**Simon task:** The correct response is indicated by the colour of a cue (or alternatively a letter), which is presented either to the left or right of a central fixation cross. In incongruent trials, the correct response is different to the spatial position of the cue (e.g. a cue indicating a right hand response is shown on the left side of the screen).

**Stop-signal reaction time task:** In a subset of trials an already initiated response (indicated by a Go-cue) has to be aborted when a visual or auditory Stop-signal is presented. The delay between the Go-cue and the Stop-signal is adjusted over time in a stair-case procedure so that patients are able to successfully stop the response in 50% of trials. The stop signal reaction time can then be computed by subtracting the mean stop signal delay from the mean reaction time.

**Stroop task:** The names of colours are written in incongruent ink colours. Participants have to state aloud the ink colours ignoring the written words (e.g. if the word ‘red’ is written in yellow ink the correct response is ‘yellow’).

In tasks with conflicting information, such as the Eriksen-Flanker task^82^ (Box 1), a strong, short-lasting cue-induced theta power increase can be observed in the STN and over medial prefrontal cortex in EEG.^38,72,83-85^ An increase in STN theta power can also be detected during deliberation in the moving dots task even if there is no clear cue onset.^18,86^ Similar to STN beta activity, STN theta power has consistently been shown to correlate with single-trial adjustments in decision thresholds.^18,69,73,86^ However, unlike STN beta power, this relationship is dependent on task context. Increases in STN theta activity only correlate with increased decision thresholds when participants are more cautious, weighing accuracy over speed^18,69,73,86^ (Fig. 2D). Relatedly, the experience of decision conflict—signalling the need for further evidence accumulation to arrive at an accurate decision—is related to increases in cortical and STN theta activity.^38,83,86,87^ These patterns are similarly observed in healthy humans in simultaneous functional MRI (fMRI) and EEG studies linking conflict-induced increases in midfrontal cortical theta in EEG to STN signals in fMRI and decision threshold adjustments.^88^ Thus, beyond their spectral properties, STN theta and beta oscillations also seem to differ regarding their modulation by task context and strategy.

What might be the role of the recorded task-related LFP modulations in the STN? LFPs reflect a summation of various underlying neural processes including synaptic transmembrane currents, calcium flux and action potentials from thousands of neurons in proximity of the recording electrode.^89^ Thus, LFP recordings can reveal synchronized, oscillatory neural population activity. A prominent theory proposes that such neural oscillations provide a ‘window in time’ where neural communication between spatially distinct brain regions can take place.^90,91^ According to this theory, the effect of action potentials on their target neurons depends on the phase of ongoing oscillations so that neurons which are aligned regarding their phases can communicate more efficiently compared to neurons that are out of sync. Several studies have demonstrated that spiking activity in the STN is aligned to the phase of theta and beta oscillations.^92-95^ In the study described above,^69^ during the moving dots task, activity changes in the beta band were predominantly observed over motor cortical areas, and in the theta band over midline prefrontal cortex (cortical signals were measured using EEG), in line with previous studies^18,83,86,88,96,97^ (Fig. 2E and F). However, beta oscillations are also expressed in prefrontal cortex^98-101^ and theta oscillations in motor cortical areas,^102,103^ arguing against a clear-cut distinction in regionally specific cortico-STN communication according to distinct frequency bands. Nevertheless, oscillatory communication might facilitate adapting which cortical areas primarily impose activity changes in the STN to mediate action control in specific time windows. For beta activity, it has been demonstrated that increased beta power at the cortical level is locked to increased beta activity at arm muscles during static contractions, which is attenuated during phasic movements.^104,105^ Thus, this neural signal could facilitate stabilization of the current posture for delaying dynamic movement.^106^ One challenge for this hypothesis is that oscillatory synchronization might be too slow to implement a quick action pause, at least for lower frequencies in the theta and beta band during outright stopping.^107^ Stopping-related single neuron activity in (primarily ventro-medial) STN occur well before any oscillatory beta increases.^42^ Thus, there is strong correlative evidence for a role of STN theta and beta oscillations in delaying actions, but does the STN have causal behavioural effects in humans? One possibility for testing this is to apply electrical stimulation to the STN using DBS and measure the resulting changes in behaviour.

## Shaping behaviour with deep brain stimulation

Building on results from rodent studies implicating STN in cognitive control,^108,109^ early evidence that STN DBS might impair functions related to cognitive control in humans came from clinical assessments after DBS surgery showing that DBS worsens performance of the Stroop task^110^ (Box 1), which is often part of the standard neuropsychological evaluation.^57,111-113^ A shortcoming of such clinical neuropsychological assessments is that often only a summary statistic is reported, e.g. the difference in reaction times in trials with versus without conflict, accuracy rates or similar. However, the proposed computational role of the STN makes a much clearer prediction. STN DBS should lead to an increase in particularly fast erroneous responses slipping through control during response conflict, while this should not be the case if responses have been slowed down.^44,45,47^ Three separate studies tested PD patients during a Simon task^114^ (Box 1) on and off DBS and analysed accuracy rates as a function of reaction times. Two of these studies found that indeed STN DBS significantly increased error rates in the fastest, but not in slower, conflict trials,^115,116^ while the third study found a similar, albeit not significant effect, putatively due to a lower sample size.^117^ Patients’ ability to avoid hasty choices during decision-making has been assessed in several studies on and off DBS. These studies have consistently observed that continuous high-frequency DBS impairs patients’ ability to slow down responses when caution is warranted, leading to reduced decision thresholds in both value-based and perceptual decision-making.^83,118-122^

There are, however, some short-comings of the approach comparing separate on versus off DBS conditions. DBS is used as a clinical treatment for PD. When patients who are chronically implanted with DBS are withdrawn from DBS treatment in one condition, their clinical state will deteriorate considerably compared to on DBS. Thus, patients are overall slower, might have tremor and experience other discomfort due to symptom exacerbation. Such more unspecific (i.e. not task-related) effects of DBS are difficult to account for in statistical designs and can introduce substantial variability. For example, for outright stopping during the stop-signal reaction time task^123^ (Box 1), previous studies have shown improvement,^124-126^ deterioration^127,128^ and no overall effects^37,129,130^ of STN DBS, despite strong evidence for causal effects of STN on outright stopping in rodent studies.^131,132^ This could, next to differences in exact electrode placement and methodologies,^127,133-135^ be due to differences in the clinical state when patients are studied on and off DBS. PD patients who express impaired response inhibition OFF treatment^136^ might show improved performance with DBS, while the opposite might be true for patients with preserved inhibition without treatment.^130^ In addition, a study showing positive effects of STN DBS on stopping in PD patients reported that this effect could also be achieved with DBS of the ventral-intermediate nucleus of the thalamus, a target for tremor-dominant PD, suggesting general DBS effects related to clinical improvement.^126^ A second limitation of classical on versus off DBS studies is that there can be ambiguity regarding the mechanism being affected by DBS, since stimulation is given continuously. For example, altered performance could be related to changes during deliberation, action preparation or outcome evaluation.^68^

To address these issues, recent studies have leveraged technical advances in DBS for applying stimulation in short bursts rather than continuously. Since the exact timing of stimulation can be varied from trial to trial, this makes any immediate changes in the clinical state of the patients unlikely (on- and off-DBS trials are compared within the same session, see later) and can facilitate inference of timing-specific effects of STN DBS on distinct processes underlying the observed behaviour.

**Figure 3:**
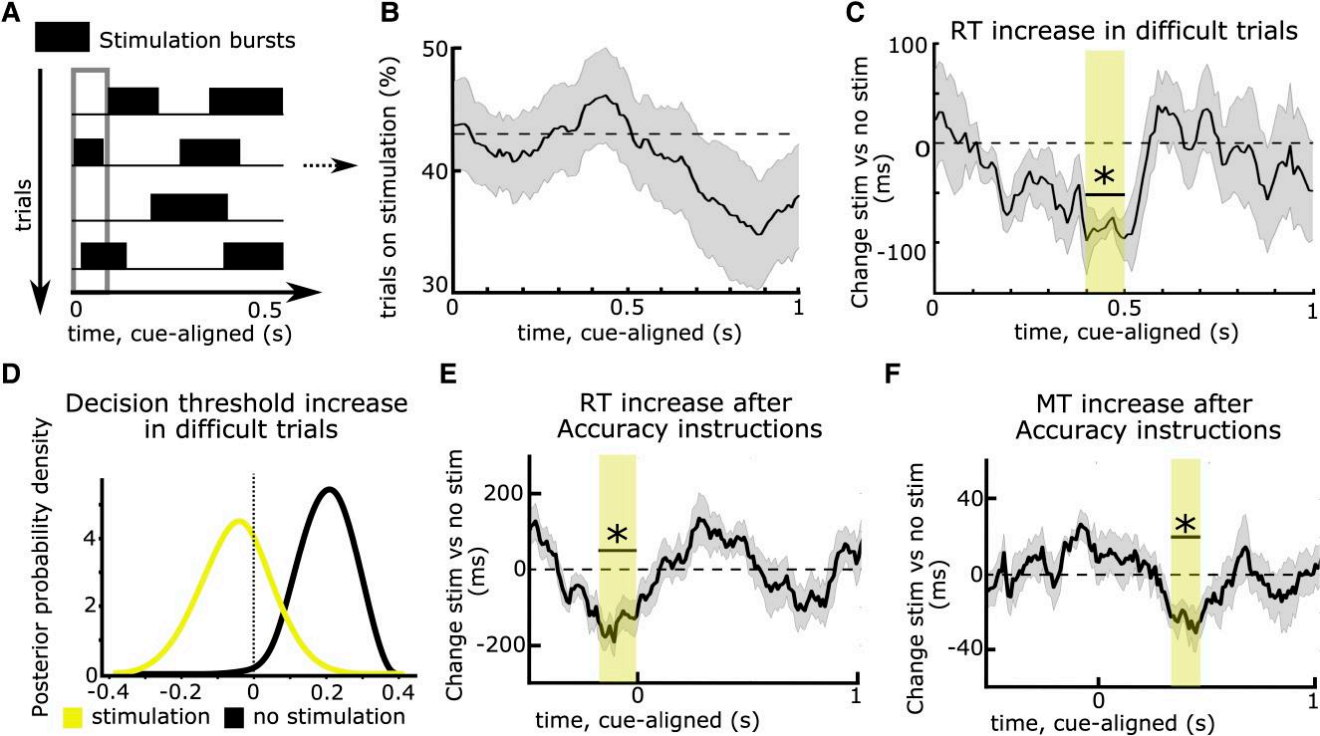
**Timing-specific stimulation of the subthalamic nucleus.** (**A**) Deep brain stimulation (DBS) can be given in bursts rather than continuously, resulting in stimulation being applied in some trials, while no stimulation occurs in other trials in any given time window (in the 100 ms time window indicated by the grey rectangle, DBS was given in trials 2 and 4, while no DBS was given in trials 1 and 3 in this example). (**B**) Using an adaptive DBS set-up, stimulation was given on average in 43% of trials (dotted line) across time windows, which varied slightly according to fluctuation in subthalamic nucleus (STN) beta activity. (**C**) DBS reduced the reaction time (RT) increase in difficult trials only when given in a specific time window, 0.4–0.5 s after onset of the moving dots cue (marked by the yellow rectangle and an asterisk). (**D**) When DBS was given in this time window, patients no longer increased their decision thresholds during more difficult trials (yellow probability distribution with a mean close to 0), while this was preserved when DBS was not given in this exact window (black probability distribution shifted to the right from 0). The same effects were observed when comparing continuous DBS to an off DBS condition (not shown). (**E**) DBS impaired patients’ abilities to properly increase their RT after Accuracy instructions when given in a time window just before onset of the moving dots cue (marked by the yellow rectangle and an asterisk). (**F**) The same as **E** but for movement times (MT). Here, the significant time window showing timing-specific effects of DBS occurred later during the trial and the DBS effects on, respectively, RT and MT were not correlated. Note that in **C** and **D**, task difficulty changed on a trial-by-trial basis, while in **E** and **F**, Speed versus Accuracy instructions changed at each trial. **A**–**D** are based on Herz *et al*.^17^**E** and **F** are based on Herz *et al*.^73^

The first study assessing timing-specific effects of STN DBS on delaying actions tested PD patients who performed a moving dots task whilst STN DBS was applied in an adaptive DBS (aDBS) design.^17^ Recorded STN beta power triggered DBS whenever STN beta power crossed a certain threshold and was turned off again when beta power fell below this threshold,^137,138^ resulting in repetitive bursts of stimulation throughout the task (Fig. 3A). Importantly, this resulted in stimulation occurring in approximately half of all trials in any 100 ms window throughout the task (Fig. 3B), so that behaviour at each time window could be compared for trials where DBS had been given versus trials where no stimulation had been given. Results showed that both aDBS and continuous DBS impaired patients’ ability to slow down responses in line with previous studies.^83,118-120^ However, during aDBS this effect was restricted to a short ∼100 ms time window during deliberation (Fig. 3C). Stimulation during this processing window abolished the dynamic increase in decision thresholds during more difficult trials observed off DBS—and even during aDBS if stimulation was not given in this specific window (Fig. 3D). Together, this study demonstrated that STN effects on decision threshold adjustments are highly dynamic and related to short processing windows. They are also consistent with the dynamics of detailed neural circuit models of STN, in which initial brief STN activation is related to a temporary pause in action selection, implementing a collapsing decision boundary.^47,139,140^

Such timing-specific effects of STN DBS have also been observed during the Stroop task.^78^ Since STN DBS was given in bursts throughout the experiment, the behavioural effect of DBS could not be explained by changes in the clinical state of the patients. What remained unclear, however, was whether the effect of DBS might have been related to fluctuations in STN beta activity, since aDBS was triggered by beta power, despite several control analyses to exclude this possibility.^17^ Furthermore, experimental factors modulating deliberation also affect how movements are performed.^141,142^ Could the observed effects of DBS on delaying choices be related to its effect on speeding up movement? To address these issues, a recent study modified stimulation so that the duration of DBS bursts and the interval between bursts were optimized to apply DBS on 50% of trials in any 100 ms window without this being triggered by changes in STN beta power. Patients performed a moving dots task in which, at each trial, patients were instructed to be as accurate or as fast as possible, which has been shown to significantly modulate both reaction times and movement speed.^141^ Movement speed was assessed by recording patients’ responses on a dynamometer. The main finding of the study was that DBS interfered with patients’ abilities to both slow down reaction times and movement speed when caution was warranted but that these effects occurred at different time points during the trial. While response times (and decision thresholds) were modulated by STN DBS just around onset of the moving dots cue (Fig. 3E), movement speed was modulated several hundred milliseconds later (Fig. 3F), and the two effects were not correlated. This demonstrated that decision and movement speed in the STN are controlled independently even if both are modified, e.g. due to time pressure. Finally, by applying stimulation bursts independently to the right versus left STN in a separate session, the study showed that STN DBS only interferes with slowing down choices when applied contralaterally to the moving hand, while ipsilateral DBS had no effects on behaviour. This finding adds to recent evidence that while cortico-STN networks can suppress behaviour broadly, i.e. beyond the task-relevant effector muscles,^37,38^ this effect is restricted to the contralateral body side.^143,144^

The two approaches described above, i.e. recording LFPs and applying stimulation through DBS electrodes, can also be combined. Using a bipolar montage surrounding the stimulation contact for common-mode rejection and appropriate artefact correction procedures, it is possible to record STN LFPs during DBS.^17,68,73,145,146^ Intriguingly, studies applying bursts of STN DBS and recording LFPs have consistently observed that timing-specific causal effects of DBS occur just prior to time windows where modulations of STN LFPs are reflective of this behaviour.^17,68,73^ For example, in the study discussed above,^17^ STN beta power from 500–800 ms after the moving dots cue correlated with decision threshold adjustments during difficult trials, with higher beta power being related to higher decision thresholds. Applying stimulation just prior to this time window, 400–500 ms after the cue, impaired patients ability to increase decision thresholds during difficult trials and reduced STN beta power in the time window where it usually (i.e. off DBS) was reflective of decision threshold adjustments. What could be the reason for this shift in timing between STN DBS effects and STN LFP correlates of behaviour? One possibility is that it might be necessary to suppress oscillations early on to prevent their expression, assuming a causal relationship between modulations in oscillatory STN activity and behavioural changes. In line with this, DBS reduces beta power longer than the exact duration of stimulation.^68,73^ Another possibility is that LFP changes are reflective of a neural process that has already taken place. For example, during outright stopping, inhibition might be achieved by modulation of fast single unit dynamics in the STN, while later occurring increases in STN beta activity might primarily maintain this state or regulate evaluative processes.^42^ A related possibility is that the relationship between oscillatory field activity and spiking might change with the delay after stimulus onset, as suggested by recent biophysical modelling of STN.^139^ Irrespective of the exact mechanism, together, these results provide strong evidence that cortico-STN circuits causally contribute to the controlled delay of action and that this dynamic process occurs in ‘critical’ time windows, which can be disturbed by DBS. This raises the question of whether STN DBS, beyond its beneficial effect on motor symptoms in PD, might also have detrimental effects rendering patients susceptible to impulsive behaviour.

## Clinical implications

**Figure 4:**
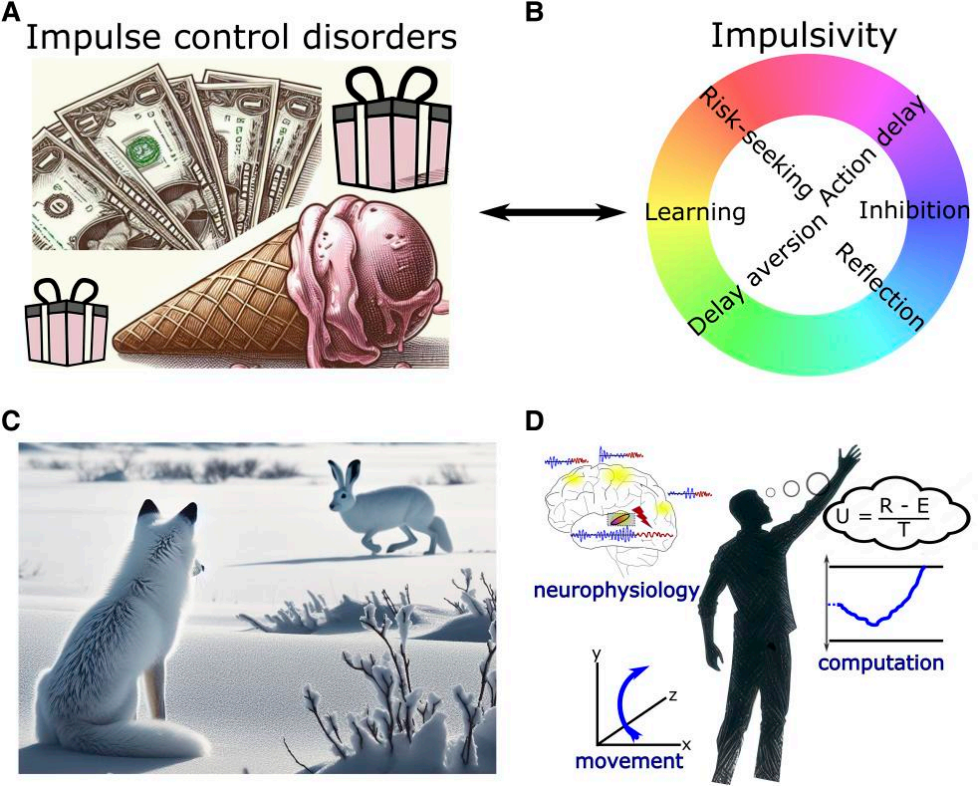
**Clinical implications and outlook.** (**A**) Impulse control disorders (ICD) are defined as pathological gambling, compulsive shopping, eating and hypersexuality along with other abnormal behaviour such as punding, hobbyism and overuse of dopaminergic medication. (**B**) Spectra of impulsivity comprise distinct underlying mechanisms. (**C**) In naturalistic behaviour, there is often no clear-cut distinction between functional categories such decision-making, perception and motor control. (**D**) Illustration of a holistic research approach combining multidimensional measures of brain activity and movement as well as computational analyses of the observed behaviour. In this example, evidence for different options is integrated regarding their utility (U) given by reward (R) subtracted by effort costs (**E**) and divided by total time (T) to obtain the reward.

At first glance, there seems to be a discrepancy between the consistently observed DBS-induced impairment in delaying choices in experimental paradigms and the relatively rare occurrence of impulse control disorders (ICD) after STN DBS.^147-149^ ICD are defined as pathological gambling, compulsive shopping, eating and sexual behaviour along with other abnormal behaviour such as punding, hobbyism and overuse of dopaminergic medication. ICD affect up to ∼25% of medicated PD patients^64,148^ (Fig. 4A). The prevalence is even higher when considering more mildly affected patients who express impulse control behaviours, which do not sufficiently impact social or occupational function to classify as a disorder.^150^ Thus, rather than reflecting a categorical (yes/no) entity, impairments in impulse control represent a spectrum of changes in behaviour, which can have a devastating impact on patients’ and their caregivers’ lives when severely expressed. The occurrence of ICD has mainly been linked to dopaminergic treatment, with a particularly high risk of symptom occurrence when using dopamine agonists.^65,148,150^ While some patients develop post-operative *de novo* ICD after STN DBS, ICD mostly improve after surgery, presumably due to a reduction in dopaminergic medication.^147,149,151^ In other words, since dopamine treatment is thought to be the main underlying cause of ICD in PD patients, any reduction of dopamine medication—e.g. when motor impairment is alleviated by STN DBS—will reduce the expression of impulse control behaviours. However, if STN DBS disturbs patients’ abilities to delay choices, as discussed above, thus rendering them more susceptible to deciding more impulsively, why would STN DBS not itself lead to the emergence of ICD?

An important aspect to consider for understanding the relationship between an impairment in delaying actions and the development of ICD is the multifaceted nature of impulsivity and its underlying mechanisms^119,122,152,153^ (Fig. 4B). In the paradigms reviewed above, patients mainly show ‘motor impulsivity’, i.e. an inability to hold back an action.^154^ In contrast to natural environments where decisions often are continuous and extend over time,^155^ in laboratory settings choices are almost always terminated by an action. Thus, motor impulsivity can lead to quicker, uncorrectable choices at lower levels of evidence. This is sometimes termed ‘reflection impulsivity’^154^ but is arguably closely related to motor impulsivity in laboratory settings.^119^ Importantly, despite the evidence considered for such hasty decisions being more strongly affected by noise, the associated choices are not necessarily more risk-prone or delay-aversive, two main aspects of ‘decision impulsivity’.^154^ Indeed, mechanistic computational models suggest that impulsivity related to risky decision-making and pathological gambling arises from imbalances of striatal dopamine altering the relative sensitivity to benefits versus costs of decisions,^156^ which is affected by dopaminergic medication but not by DBS.^119,157^ Thus, dopamine, but not STN DBS, can render PD patients more sensitive to possible benefits and less sensitive to potential costs of choices, and STN DBS, but not dopamine, reduces deliberation time, resulting in more hasty decisions.^122,153^ If anything, deciding faster might make patients fall back on habitual choices^158,159^ and PD patients tend to be habitually risk averse.^160,161^ With few exceptions,^162,163^ STN DBS has been shown to have no significant effects on patients’ decisions when these are less closely related to the speed of deliberation, e.g. when choosing between smaller immediate versus larger delayed rewards^164-167^ or learning from negative feedback.^119,164,168,169^

Together, an impairment in delaying choices due to STN DBS might render people more susceptible to initiate behaviour without proper forethought, e.g. getting up very quickly from a chair despite postural instability.^119^ However, this mechanism is different from increased reward delay aversion, reduced risk aversion or impairments in negative feedback learning, core processes thought to underlie the development of compulsive behaviours in clinical ICD, which are mainly related to dopaminergic medication.^64,148,150,157,170-172^

Is it possible to improve motor impairment in PD with STN DBS without simultaneously affecting motor impulsivity? In a hypothetical model of cortico-basal ganglia networks in which decision-making circuits are segregated from movement control, it might be possible to target beta oscillatory activity to specifically affect motor processes leaving decision-making processes unaltered. However, such a model seems incompatible with current views of brain organization and function. Imagine a fox hunting for prey (Fig. 4C). To successfully gather food, the fox needs to e.g. perceive a white hare against a white snowy background, stalk the hare to keep the target in its visual field and decide when and how to attack. In such naturalistic environments, it is impossible to clearly distinguish between decision-making and motor control, since both are continuous and inextricably intertwined.^155^ Such interactions are also implicated even in simple cognitive studies of task switching, wherein uncertainty about the latent task set rule in prefrontal cortex triggers an STN pause in the motor circuit, preventing premature responses and reducing task-set interference.^173^ According to this framework, even disrupting STN solely at the motor level will then impair decision-making and increase task-set interference.

Indeed, most, if not all, neural areas involved in decision-making modify and reflect movement^174^ and evolving decisions can be tracked in brain networks classified as motor.^175,176^ In DBS studies, decision threshold adjustments can be linked to STN activity changes both in the theta and beta range.^10,17,69,73,86^ Thus, to some extent, interfering with brain networks for therapy will always have diverse effects due to the multiple functionalities of brain organization (the same network can implement multiple functions).^177^ Nevertheless, on a more pragmatic level, there is good evidence that progress in spatially-focused DBS as well as adaptive DBS, which only gives stimulation when deemed necessary based on feedback markers, can improve clinical efficacy and spare side effects.^178-180^ To further advance this exciting field of research, it will be vital to better understand where and when DBS should be applied to optimize effects on abnormal neural activity patterns, even if it might remain fictitious to exclusively modulate one specific behaviourally-defined variable.

### Outlook

In this review, we focused on the role of cortico-subthalamic circuits in the controlled delay of actions. However, cortico-basal ganglia circuits subserve a variety of other important functions, such as movement invigoration, action selection and learning.^26,28,181,182^ Many of these behaviourally defined functions are closely interrelated (e.g. options with higher utility should be more likely to be selected and also be acquired more vigorously)^183^ and therefore putatively rely on similar or even common neural mechanisms. This could be leveraged for therapy aiming to reinstate functionality that is impaired in neurological disorders such as PD. To this end it will be important to carefully map the effects of DBS on well-defined behavioural changes and their underlying computations. Optimally, this will involve multidimensional neurophysiological measures beyond a single frequency band or recording site,^184^ precisely controlled stimulation patterns and behavioural measures, including body movement, in studies that are grounded in clear theoretical frameworks allowing specific predictions to be tested^106^ (Fig. 4D). Since cortico-basal ganglia dysfunction has been implied in a wide range of neurological and psychiatric diseases, the results discussed here might also be useful for the study of other brain disorders and could inform future DBS study designs.^180^ For example, DBS of the ventro-medial STN is currently being investigated for obsessive-compulsive disorders,^185^ and a better mechanistic understanding of DBS effects on action control in obsessive-compulsive disorders could advance current treatment strategies. Novel sensing, directional DBS devices^51^ offer the unique possibility to translate insights from studies using DBS as an experimental tool to clinical therapy, bridging the gap between bench and bedside.

## References

[1] Adams RA , Shipp S, Friston KJ. Predictions not commands: Active inference in the motor system. Brain Struct Funct. 2013;218:611–643. 23129312 10.1007/s00429-012-0475-5 PMC3637647

[2] Musslick S , Cohen JD. Rationalizing constraints on the capacity for cognitive control. Trends Cogn Sci. 2021;25:757–775. 34332856 10.1016/j.tics.2021.06.001

[3] Shenhav A , Cohen JD, Botvinick MM. Dorsal anterior cingulate cortex and the value of control. Nat Neurosci. 2016;19:1286–1291. 27669989 10.1038/nn.4384

[4] Balleine BW , O'Doherty JP. Human and rodent homologies in action control: Corticostriatal determinants of goal-directed and habitual action. Neuropsychopharmacology. 2010;35:48–69. 19776734 10.1038/npp.2009.131 PMC3055420

[5] Drummond N , Niv Y. Model-based decision making and model-free learning. Curr Biol. 2020;30:R860–R865. 32750340 10.1016/j.cub.2020.06.051

[6] Friedman NP , Robbins TW. The role of prefrontal cortex in cognitive control and executive function. Neuropsychopharmacology. 2022;47:72–89. 34408280 10.1038/s41386-021-01132-0 PMC8617292

[7] Manohar SG , Chong TT, Apps MA, et al Reward pays the cost of noise reduction in motor and cognitive control. Curr Biol. 2015;25:1707–1716. 26096975 10.1016/j.cub.2015.05.038 PMC4557747

[8] Westbrook A , Frank MJ, Cools R. A mosaic of cost-benefit control over cortico-striatal circuitry. Trends Cogn Sci. 2021;25:710–721. 34120845 10.1016/j.tics.2021.04.007 PMC8282630

[9] Glimcher PW , Dorris MC, Bayer HM. Physiological utility theory and the neuroeconomics of choice. Games Econ Behav. 2005;52:213–256. 16845435 10.1016/j.geb.2004.06.011 PMC1502377

[10] Herz DM , Bogacz R, Brown P. Neuroscience: Impaired decision-making in Parkinson's disease. Curr Biol. 2016;26:R671–R673. 27458912 10.1016/j.cub.2016.05.075 PMC4986883

[11] Shadlen MN , Kiani R. Decision making as a window on cognition. Neuron. 2013;80:791–806. 24183028 10.1016/j.neuron.2013.10.047 PMC3852636

[12] Bogacz R , Brown E, Moehlis J, Holmes P, Cohen JD. The physics of optimal decision making: A formal analysis of models of performance in two-alternative forced-choice tasks. Psychol Rev. 2006;113:700–765. 17014301 10.1037/0033-295X.113.4.700

[13] Bogacz R , Wagenmakers EJ, Forstmann BU, Nieuwenhuis S. The neural basis of the speed-accuracy tradeoff. Trends Neurosci. 2010;33:10–16. 19819033 10.1016/j.tins.2009.09.002

[14] Ratcliff R , McKoon G. The diffusion decision model: Theory and data for two-choice decision tasks. Neural Comput. 2008;20:873–922. 18085991 10.1162/neco.2008.12-06-420 PMC2474742

[15] Wiecki TV , Sofer I, Frank MJ. HDDM: Hierarchical Bayesian estimation of the drift-diffusion model in python. Front Neuroinform. 2013;7:14. 23935581 10.3389/fninf.2013.00014 PMC3731670

[16] Frank MJ . Hold your horses: A dynamic computational role for the subthalamic nucleus in decision making. Neural Netw. 2006;19:1120–1136. 16945502 10.1016/j.neunet.2006.03.006

[17] Herz DM , Little S, Pedrosa DJ, et al Mechanisms underlying decision-making as revealed by deep-brain stimulation in patients with Parkinson's disease. Curr Biol. 2018;28:1169–1178.e6. 29606416 10.1016/j.cub.2018.02.057 PMC5912902

[18] Herz DM , Zavala BA, Bogacz R, Brown P. Neural correlates of decision thresholds in the human subthalamic nucleus. Curr Biol. 2016;26:916–920. 26996501 10.1016/j.cub.2016.01.051 PMC4826434

[19] Malhotra G , Leslie DS, Ludwig CJH, Bogacz R. Time-varying decision boundaries: Insights from optimality analysis. Psychon Bull Rev. 2018;25:971–996. 28730465 10.3758/s13423-017-1340-6 PMC5990589

[20] Purcell BA , Kiani R. Neural mechanisms of post-error adjustments of decision policy in parietal cortex. Neuron. 2016;89:658–671. 26804992 10.1016/j.neuron.2015.12.027 PMC4742416

[21] Bogacz R . Dopamine role in learning and action inference. Elife. 2020;9:e53262. 32633715 10.7554/eLife.53262 PMC7392608

[22] Hayden BY , Niv Y. The case against economic values in the orbitofrontal cortex (or anywhere else in the brain). Behav Neurosci. 2021;135:192–201. 34060875 10.1037/bne0000448 PMC12398331

[23] Miller KJ , Shenhav A, Ludvig EA. Habits without values. Psychol Rev. 2019;126:292–311. 30676040 10.1037/rev0000120 PMC6548181

[24] Frömer R , Shenhav A. Filling the gaps: Cognitive control as a critical lens for understanding mechanisms of value-based decision-making. Neurosci Biobehav Rev. 2022;134:104483. 34902441 10.1016/j.neubiorev.2021.12.006 PMC8844247

[25] Grillner S , Robertson B. The basal ganglia over 500 million years. Curr Biol. 2016;26:R1088–R1100. 27780050 10.1016/j.cub.2016.06.041

[26] Dudman JT , Krakauer JW. The basal ganglia: From motor commands to the control of vigor. Curr Opin Neurobiol. 2016;37:158–166. 27012960 10.1016/j.conb.2016.02.005

[27] Gerfen CR , Bolam JP. The neuroanatomical organization of the basal ganglia. In: Steiner H, Tseng KY, eds. Handbook of basal ganglia structure and function. 2nd ed. Elsevier; 2016:3–28.

[28] Redgrave P , Vautrelle N, Reynolds JN. Functional properties of the basal ganglia's re-entrant loop architecture: Selection and reinforcement. Neuroscience. 2011;198:138–151. 21821101 10.1016/j.neuroscience.2011.07.060

[29] Yin HH . The basal ganglia in action. Neuroscientist. 2017;23:299–313. 27306757 10.1177/1073858416654115 PMC6500733

[30] Alexander GE , DeLong MR, Strick PL. Parallel organization of functionally segregated circuits linking basal ganglia and cortex. Annu Rev Neurosci. 1986;9:357–381. 3085570 10.1146/annurev.ne.09.030186.002041

[31] Haynes WI , Haber SN. The organization of prefrontal-subthalamic inputs in primates provides an anatomical substrate for both functional specificity and integration: Implications for basal ganglia models and deep brain stimulation. J Neurosci. 2013;33:4804–4814. 23486951 10.1523/JNEUROSCI.4674-12.2013 PMC3755746

[32] Bevan MD . The subthalamic nucleus. In: Steiner H, Tseng KY, eds. Handbook of basal ganglia structure and function. 2nd ed. Elsevier; 2016.

[33] Nambu A , Tokuno H, Hamada I, et al Excitatory cortical inputs to pallidal neurons via the subthalamic nucleus in the monkey. J Neurophysiol. 2000;84:289–300. 10899204 10.1152/jn.2000.84.1.289

[34] Oorshot DE . Cell types in the different nuclei of the basal ganglia. In: Steiner H, Tseng KY, eds. Handbook of basal ganglia structure and function. 2nd ed. Elsevier; 2016.

[35] Hannah R , Aron AR. Towards real-world generalizability of a circuit for action-stopping. Nat Rev Neurosci. 2021;22:538–552. 34326532 10.1038/s41583-021-00485-1 PMC8972073

[36] Nambu A , Tokuno H, Takada M. Functional significance of the cortico-subthalamo-pallidal ‘hyperdirect’ pathway. Neurosci Res. 2002;43:111–117. 12067746 10.1016/s0168-0102(02)00027-5

[37] Wessel JR , Diesburg DA, Chalkley NH, Greenlee JDW. A causal role for the human subthalamic nucleus in non-selective cortico-motor inhibition. Curr Biol. 2022;32:3785–3791.e3. 35841891 10.1016/j.cub.2022.06.067 PMC9511894

[38] Wessel JR , Waller DA, Greenlee JD. Non-selective inhibition of inappropriate motor-tendencies during response-conflict by a fronto-subthalamic mechanism. Elife. 2019;8:e42959. 31063130 10.7554/eLife.42959 PMC6533064

[39] Aron AR , Herz DM, Brown P, Forstmann BU, Zaghloul K. Frontosubthalamic circuits for control of action and cognition. J Neurosci. 2016;36:11489–11495. 27911752 10.1523/JNEUROSCI.2348-16.2016 PMC5125216

[40] Bonini F , Burle B, Liégeois-Chauvel C, Régis J, Chauvel P, Vidal F. Action monitoring and medial frontal cortex: Leading role of supplementary motor area. Science. 2014;343:888–891. 24558161 10.1126/science.1247412

[41] Chen W , de Hemptinne C, Miller AM, et al Prefrontal-Subthalamic hyperdirect pathway modulates movement inhibition in humans. Neuron. 2020;106:579–588.e3. 32155442 10.1016/j.neuron.2020.02.012 PMC7274135

[42] Mosher CP , Mamelak AN, Malekmohammadi M, Pouratian N, Rutishauser U. Distinct roles of dorsal and ventral subthalamic neurons in action selection and cancellation. Neuron. 2021;109:869–881.e6. 33482087 10.1016/j.neuron.2020.12.025 PMC7933114

[43] van Wijk BCM , Alkemade A, Forstmann BU. Functional segregation and integration within the human subthalamic nucleus from a micro- and meso-level perspective. Cortex. 2020;131:103–113. 32823130 10.1016/j.cortex.2020.07.004

[44] Isoda M , Hikosaka O. Switching from automatic to controlled action by monkey medial frontal cortex. Nat Neurosci. 2007;10:240–248. 17237780 10.1038/nn1830

[45] Isoda M , Hikosaka O. Role for subthalamic nucleus neurons in switching from automatic to controlled eye movement. J Neurosci. 2008;28:7209–7218. 18614691 10.1523/JNEUROSCI.0487-08.2008 PMC2667154

[46] Redgrave P , Rodriguez M, Smith Y, et al Goal-directed and habitual control in the basal ganglia: Implications for Parkinson's disease. Nat Rev Neurosci. 2010;11:760–772. 20944662 10.1038/nrn2915 PMC3124757

[47] Wiecki TV , Frank MJ. A computational model of inhibitory control in frontal cortex and basal ganglia. Psychol Rev. 2013;120:329–355. 23586447 10.1037/a0031542

[48] Surmeier DJ , Obeso JA, Halliday GM. Parkinson's disease is not simply a prion disorder. J Neurosci. 2017;37:9799–9807. 29021297 10.1523/JNEUROSCI.1787-16.2017 PMC5637112

[49] Bloem BR , Okun MS, Klein C. Parkinson's disease. Lancet. 2021;397:2284–2303. 33848468 10.1016/S0140-6736(21)00218-X

[50] Bologna M , Paparella G, Fasano A, Hallett M, Berardelli A. Evolving concepts on bradykinesia. Brain. 2020;143:727–750. 31834375 10.1093/brain/awz344 PMC8205506

[51] Krauss JK , Lipsman N, Aziz T, et al Technology of deep brain stimulation: Current status and future directions. Nat Rev Neurol. 2021;17:75–87. 33244188 10.1038/s41582-020-00426-z PMC7116699

[52] Muthuraman M , Bange M, Koirala N, et al Cross-frequency coupling between gamma oscillations and deep brain stimulation frequency in Parkinson's disease. Brain. 2020;143:3393–3407. 33150359 10.1093/brain/awaa297 PMC7116448

[53] Neumann WJ , Steiner LA, Milosevic L. Neurophysiological mechanisms of deep brain stimulation across spatiotemporal resolutions. Brain. 2023;146:4456–4468. 37450573 10.1093/brain/awad239 PMC10629774

[54] Koirala N , Fleischer V, Glaser M, et al Frontal lobe connectivity and network community characteristics are associated with the outcome of subthalamic nucleus deep brain stimulation in patients with Parkinson's disease. Brain Topogr. 2018;31:311–321. 28986718 10.1007/s10548-017-0597-4

[55] Grill WM , Snyder AN, Miocinovic S. Deep brain stimulation creates an informational lesion of the stimulated nucleus. Neuroreport. 2004;15:1137–1140. 15129161 10.1097/00001756-200405190-00011

[56] Drummond NM , Chen R. Deep brain stimulation and recordings: Insights into the contributions of subthalamic nucleus in cognition. Neuroimage. 2020;222:117300. 32828919 10.1016/j.neuroimage.2020.117300

[57] Jahanshahi M , Ardouin CM, Brown RG, et al The impact of deep brain stimulation on executive function in Parkinson's disease. Brain. 2000;123(Pt 6):1142–1154. 10825353 10.1093/brain/123.6.1142

[58] Ricciardi L , Apps M, Little S. Uncovering the neurophysiology of mood, motivation and behavioral symptoms in Parkinson's disease through intracranial recordings. NPJ Parkinsons Dis. 2023;9:136. 37735477 10.1038/s41531-023-00567-0 PMC10514046

[59] Zavala B , Zaghloul K, Brown P. The subthalamic nucleus, oscillations, and conflict. Mov Disord. 2015;30:328–338. 25688872 10.1002/mds.26072 PMC4357561

[60] Brown P , Oliviero A, Mazzone P, Insola A, Tonali P, Di Lazzaro V. Dopamine dependency of oscillations between subthalamic nucleus and pallidum in Parkinson's disease. J Neurosci. 2001;21:1033–1038. 11157088 10.1523/JNEUROSCI.21-03-01033.2001 PMC6762327

[61] Tinkhauser G , Pogosyan A, Little S, et al The modulatory effect of adaptive deep brain stimulation on beta bursts in Parkinson's disease. Brain. 2017;140:1053–1067. 28334851 10.1093/brain/awx010 PMC5382944

[62] Olaru M , Cernera S, Hahn A, et al Motor network gamma oscillations in chronic home recordings predict dyskinesia in Parkinson's disease. Brain. 2024;147:2038–2052. 38195196 10.1093/brain/awae004 PMC11146421

[63] Wiest C , Torrecillos F, Tinkhauser G, et al Finely-tuned gamma oscillations: Spectral characteristics and links to dyskinesia. Exp Neurol. 2022;351:113999. 35143832 10.1016/j.expneurol.2022.113999 PMC7612436

[64] Antonini A , Barone P, Bonuccelli U, Annoni K, Asgharnejad M, Stanzione P. ICARUS study: Prevalence and clinical features of impulse control disorders in Parkinson's disease. J Neurol Neurosurg Psychiatry. 2017;88:317–324. 28315845 10.1136/jnnp-2016-315277

[65] Weintraub D , Koester J, Potenza MN, et al Impulse control disorders in Parkinson disease: A cross-sectional study of 3090 patients. Arch Neurol. 2010;67:589–595. 20457959 10.1001/archneurol.2010.65

[66] Cassidy M , Mazzone P, Oliviero A, et al Movement-related changes in synchronization in the human basal ganglia. Brain. 2002;125(Pt 6):1235–1246. 12023312 10.1093/brain/awf135

[67] Fischer P , Chen CC, Chang YJ, et al Alternating modulation of subthalamic nucleus beta oscillations during stepping. J Neurosci. 2018;38:5111–5121. 29760182 10.1523/JNEUROSCI.3596-17.2018 PMC5977446

[68] Herz DM , Bange M, Gonzalez-Escamilla G, et al Dynamic modulation of subthalamic nucleus activity facilitates adaptive behavior. PLoS Biol. 2023;21:e3002140. 37262014 10.1371/journal.pbio.3002140 PMC10234560

[69] Herz DM , Tan H, Brittain JS, et al Distinct mechanisms mediate speed-accuracy adjustments in cortico-subthalamic networks. Elife. 2017;6:e21481. 28137358 10.7554/eLife.21481 PMC5287713

[70] Oswal A , Litvak V, Brücke C, et al Cognitive factors modulate activity within the human subthalamic nucleus during voluntary movement in Parkinson's disease. J Neurosci. 2013;33:15815–15826. 24089489 10.1523/JNEUROSCI.1790-13.2013 PMC3787500

[71] Tan H , Zavala B, Pogosyan A, et al Human subthalamic nucleus in movement error detection and its evaluation during visuomotor adaptation. J Neurosci. 2014;34:16744–16754. 25505327 10.1523/JNEUROSCI.3414-14.2014 PMC4261099

[72] Zavala B , Brittain JS, Jenkinson N, et al Subthalamic nucleus local field potential activity during the Eriksen flanker task reveals a novel role for theta phase during conflict monitoring. J Neurosci. 2013;33:14758–14766. 24027276 10.1523/JNEUROSCI.1036-13.2013 PMC3771028

[73] Herz DM , Bange M, Gonzalez-Escamilla G, et al Dynamic control of decision and movement speed in the human basal ganglia. Nat Commun. 2022;13:7530. 36476581 10.1038/s41467-022-35121-8 PMC9729212

[74] Kühn AA , Williams D, Kupsch A, et al Event-related beta desynchronization in human subthalamic nucleus correlates with motor performance. Brain. 2004;127(Pt 4):735–746. 14960502 10.1093/brain/awh106

[75] Alegre M , Lopez-Azcarate J, Obeso I, et al The subthalamic nucleus is involved in successful inhibition in the stop-signal task: A local field potential study in Parkinson's disease. Exp Neurol. 2013;239:1–12. 22975442 10.1016/j.expneurol.2012.08.027

[76] Benis D , David O, Lachaux JP, et al Subthalamic nucleus activity dissociates proactive and reactive inhibition in patients with Parkinson's disease. Neuroimage. 2014;91:273–281. 24368260 10.1016/j.neuroimage.2013.10.070

[77] Brittain JS , Watkins KE, Joundi RA, et al A role for the subthalamic nucleus in response inhibition during conflict. J Neurosci. 2012;32:13396–13401. 23015430 10.1523/JNEUROSCI.2259-12.2012 PMC3492743

[78] Ghahremani A , Aron AR, Udupa K, et al Event-related deep brain stimulation of the subthalamic nucleus affects conflict processing. Ann Neurol. 2018;84:515–526. 30152889 10.1002/ana.25312 PMC6431255

[79] Ray NJ , Brittain JS, Holland P, et al The role of the subthalamic nucleus in response inhibition: Evidence from local field potential recordings in the human subthalamic nucleus. Neuroimage. 2012;60:271–278. 22209815 10.1016/j.neuroimage.2011.12.035

[80] Wessel JR , Ghahremani A, Udupa K, et al Stop-related subthalamic beta activity indexes global motor suppression in Parkinson's disease. Mov Disord. 2016;31:1846–1853. 27474845 10.1002/mds.26732 PMC5154922

[81] Newsome WT , Paré EB. A selective impairment of motion perception following lesions of the middle temporal visual area (MT). J Neurosci. 1988;8:2201–2211. 3385495 10.1523/JNEUROSCI.08-06-02201.1988 PMC6569328

[82] Eriksen BA , Eriksen CW. Effects of noise letters upon the identification of a target letter in a nonsearch task. Percept Psychophys. 1974;16:143–149.

[83] Cavanagh JF , Wiecki TV, Cohen MX, et al Subthalamic nucleus stimulation reverses mediofrontal influence over decision threshold. Nat Neurosci. 2011;14:1462–1467. 21946325 10.1038/nn.2925 PMC3394226

[84] Hell F , Taylor PCJ, Mehrkens JH, Bötzel K. Subthalamic stimulation, oscillatory activity and connectivity reveal functional role of STN and network mechanisms during decision making under conflict. Neuroimage. 2018;171:222–233. 29307607 10.1016/j.neuroimage.2018.01.001

[85] Zavala B , Tan H, Ashkan K, et al Human subthalamic nucleus-medial frontal cortex theta phase coherence is involved in conflict and error related cortical monitoring. Neuroimage. 2016;137:178–187. 27181763 10.1016/j.neuroimage.2016.05.031 PMC4927260

[86] Zavala BA , Tan H, Little S, et al Midline frontal cortex low-frequency activity drives subthalamic nucleus oscillations during conflict. J Neurosci. 2014;34:7322–7333. 24849364 10.1523/JNEUROSCI.1169-14.2014 PMC4028502

[87] Zeng K , Drummond NM, Ghahremani A, et al Fronto-subthalamic phase synchronization and cross-frequency coupling during conflict processing. Neuroimage. 2021;238:118205. 34077804 10.1016/j.neuroimage.2021.118205 PMC8944202

[88] Frank MJ , Gagne C, Nyhus E, et al fMRI and EEG predictors of dynamic decision parameters during human reinforcement learning. J Neurosci. 2015;35:485–494. 25589744 10.1523/JNEUROSCI.2036-14.2015 PMC4293405

[89] Buzsáki G , Anastassiou CA, Koch C. The origin of extracellular fields and currents–EEG, ECoG, LFP and spikes. Nat Rev Neurosci. 2012;13:407–420. 22595786 10.1038/nrn3241 PMC4907333

[90] Fries P . A mechanism for cognitive dynamics: Neuronal communication through neuronal coherence. Trends Cogn Sci. 2005;9:474–480. 16150631 10.1016/j.tics.2005.08.011

[91] Fries P . Rhythms for cognition: Communication through coherence. Neuron. 2015;88:220–235. 26447583 10.1016/j.neuron.2015.09.034 PMC4605134

[92] Kühn AA , Trottenberg T, Kivi A, Kupsch A, Schneider GH, Brown P. The relationship between local field potential and neuronal discharge in the subthalamic nucleus of patients with Parkinson's disease. Exp Neurol. 2005;194:212–220. 15899258 10.1016/j.expneurol.2005.02.010

[93] Meidahl AC , Moll CKE, van Wijk BCM, et al Synchronised spiking activity underlies phase amplitude coupling in the subthalamic nucleus of Parkinson's disease patients. Neurobiol Dis. 2019;127:101–113. 30753889 10.1016/j.nbd.2019.02.005 PMC6545172

[94] Scherer M , Steiner LA, Kalia SK, et al Single-neuron bursts encode pathological oscillations in subcortical nuclei of patients with Parkinson's disease and essential tremor. Proc Natl Acad Sci U S A. 2022;119:e2205881119. 36018837 10.1073/pnas.2205881119 PMC9436336

[95] Zavala B , Damera S, Dong JW, Lungu C, Brown P, Zaghloul KA. Human subthalamic nucleus theta and beta oscillations entrain neuronal firing during sensorimotor conflict. Cereb Cortex. 2017;27:496–508. 26494798 10.1093/cercor/bhv244 PMC5390852

[96] Horn A , Neumann WJ, Degen K, Schneider GH, Kühn AA. Toward an electrophysiological “sweet spot” for deep brain stimulation in the subthalamic nucleus. Hum Brain Mapp. 2017;38:3377–3390. 28390148 10.1002/hbm.23594 PMC6867148

[97] Rodriguez-Oroz MC , López-Azcárate J, Garcia-Garcia D, et al Involvement of the subthalamic nucleus in impulse control disorders associated with Parkinson's disease. Brain. 2011;134(Pt 1):36–49. 21059746 10.1093/brain/awq301

[98] Jana S , Hannah R, Muralidharan V, Aron AR. Temporal cascade of frontal, motor and muscle processes underlying human action-stopping. Elife. 2020;9:e50371. 32186515 10.7554/eLife.50371 PMC7159878

[99] Schaum M , Pinzuti E, Sebastian A, et al Right inferior frontal gyrus implements motor inhibitory control via beta-band oscillations in humans. Elife. 2021;10:e61679. 33755019 10.7554/eLife.61679 PMC8096430

[100] Schmidt R , Herrojo Ruiz M, Kilavik BE, Lundqvist M, Starr PA, Aron AR. Beta oscillations in working memory, executive control of movement and thought, and sensorimotor function. J Neurosci. 2019;39:8231–8238. 31619492 10.1523/JNEUROSCI.1163-19.2019 PMC6794925

[101] Zavala B , Jang A, Trotta M, Lungu CI, Brown P, Zaghloul KA. Cognitive control involves theta power within trials and beta power across trials in the prefrontal-subthalamic network. Brain. 2018;141:3361–3376. 30358821 10.1093/brain/awy266 PMC6272116

[102] Pellegrino G , Tomasevic L, Herz DM, Larsen KM, Siebner HR. Theta activity in the left dorsal premotor cortex during action re-evaluation and motor reprogramming. Front Hum Neurosci. 2018;12:364. 30297991 10.3389/fnhum.2018.00364 PMC6161550

[103] Zhang Q , Zhao B, Neumann WJ, et al Low-frequency oscillations link frontal and parietal cortex with subthalamic nucleus in conflicts. Neuroimage. 2022;258:119389. 35714885 10.1016/j.neuroimage.2022.119389

[104] Echeverria-Altuna I , Quinn AJ, Zokaei N, Woolrich MW, Nobre AC, van Ede F. Transient beta activity and cortico-muscular connectivity during sustained motor behaviour. Prog Neurobiol. 2022;214:102281. 35550908 10.1016/j.pneurobio.2022.102281 PMC9742854

[105] Schoffelen JM , Oostenveld R, Fries P. Imaging the human motor system's beta-band synchronization during isometric contraction. Neuroimage. 2008;41:437–447. 18396062 10.1016/j.neuroimage.2008.01.045

[106] Herz DM , Brown P. Moving, fast and slow: Behavioural insights into bradykinesia in Parkinson's disease. Brain. 2023;146:3576–3586. 36864683 10.1093/brain/awad069 PMC10473574

[107] Fischer P , Pogosyan A, Herz DM, et al Subthalamic nucleus gamma activity increases not only during movement but also during movement inhibition. Elife. 2017;6:e23947. 28742498 10.7554/eLife.23947 PMC5526662

[108] Baunez C , Nieoullon A, Amalric M. In a rat model of Parkinsonism, lesions of the subthalamic nucleus reverse increases of reaction time but induce a dramatic premature responding deficit. J Neurosci. 1995;15:6531–6541. 7472415 10.1523/JNEUROSCI.15-10-06531.1995 PMC6578020

[109] Baunez C , Robbins TW. Bilateral lesions of the subthalamic nucleus induce multiple deficits in an attentional task in rats. Eur J Neurosci. 1997;9:2086–2099. 9421169 10.1111/j.1460-9568.1997.tb01376.x

[110] Stroop JR . Studies of interference in series verbal reactions. J Exp Psychol. 1935;18:643–662.

[111] Hamdan AC , Vieira MD. Stroop test for Parkinson's disease with deep brain stimulation: A systematic review. Innov Clin Neurosci. 2022;19(10–12):29–34. 36591546 PMC9776780

[112] Jahanshahi M , Obeso I, Baunez C, Alegre M, Krack P. Parkinson's disease, the subthalamic nucleus, inhibition, and impulsivity. Mov Disord. 2015;30:128–140. 25297382 10.1002/mds.26049

[113] Witt K , Pulkowski U, Herzog J, et al Deep brain stimulation of the subthalamic nucleus improves cognitive flexibility but impairs response inhibition in Parkinson disease. Arch Neurol. 2004;61:697–700. 15148146 10.1001/archneur.61.5.697

[114] Simon JR . Ear preference in a simple reaction-time task. J Exp Psychol. 1967;75:49–55. 6065838 10.1037/h0021281

[115] Fluchère F , Burle B, Vidal F, et al Subthalamic nucleus stimulation, dopaminergic treatment and impulsivity in Parkinson's disease. Neuropsychologia. 2018;117:167–177. 29458074 10.1016/j.neuropsychologia.2018.02.016

[116] Wylie SA , Ridderinkhof KR, Elias WJ, et al Subthalamic nucleus stimulation influences expression and suppression of impulsive behaviour in Parkinson's disease. Brain. 2010;133(Pt 12):3611–3624. 20861152 10.1093/brain/awq239 PMC2995881

[117] van Wouwe NC , Pallavaram S, Phibbs FT, et al Focused stimulation of dorsal subthalamic nucleus improves reactive inhibitory control of action impulses. Neuropsychologia. 2017;99:37–47. 28237741 10.1016/j.neuropsychologia.2017.02.016 PMC5493526

[118] Coulthard EJ , Bogacz R, Javed S, et al Distinct roles of dopamine and subthalamic nucleus in learning and probabilistic decision making. Brain. 2012;135(Pt 12):3721–3734. 23114368 10.1093/brain/aws273 PMC3525052

[119] Frank MJ , Samanta J, Moustafa AA, Sherman SJ. Hold your horses: Impulsivity, deep brain stimulation, and medication in parkinsonism. Science. 2007;318:1309–1312. 17962524 10.1126/science.1146157

[120] Green N , Bogacz R, Huebl J, Beyer AK, Kühn AA, Heekeren HR. Reduction of influence of task difficulty on perceptual decision making by STN deep brain stimulation. Curr Biol. 2013;23:1681–1684. 23932401 10.1016/j.cub.2013.07.001

[121] Pote I , Torkamani M, Kefalopoulou ZM, et al Subthalamic nucleus deep brain stimulation induces impulsive action when patients with Parkinson's disease act under speed pressure. Exp Brain Res. 2016;234:1837–1848. 26892884 10.1007/s00221-016-4577-9 PMC4893074

[122] Pagnier G , Asaad WF, Frank MJ. Double dissociation of dopamine and subthalamic nucleus stimulation on effortful cost/benefit decision making. Curr Biol. 2024;34:655–660.e3. 38183986 10.1016/j.cub.2023.12.045 PMC10872531

[123] Logan GD , Cowan WB. On the ability to inhibit thought and action: A theory of an act of control. Psychol Rev. 1984;91:295–327. 10.1037/a0035230 24490789

[124] Mirabella G , Iaconelli S, Romanelli P, et al Deep brain stimulation of subthalamic nuclei affects arm response inhibition in Parkinson's patients. Cereb Cortex. 2012;22:1124–1132. 21810782 10.1093/cercor/bhr187

[125] Swann N , Poizner H, Houser M, et al Deep brain stimulation of the subthalamic nucleus alters the cortical profile of response inhibition in the beta frequency band: A scalp EEG study in Parkinson's disease. J Neurosci. 2011;31:5721–5729. 21490213 10.1523/JNEUROSCI.6135-10.2011 PMC3086079

[126] van den Wildenberg WP , van Boxtel GJ, van der Molen MW, Bosch DA, Speelman JD, Brunia CH. Stimulation of the subthalamic region facilitates the selection and inhibition of motor responses in Parkinson's disease. J Cogn Neurosci. 2006;18:626–636. 16768365 10.1162/jocn.2006.18.4.626

[127] Lofredi R , Auernig GC, Irmen F, et al Subthalamic stimulation impairs stopping of ongoing movements. Brain. 2021;144:44–52. 33253351 10.1093/brain/awaa341

[128] Obeso I , Wilkinson L, Rodríguez-Oroz MC, Obeso JA, Jahanshahi M. Bilateral stimulation of the subthalamic nucleus has differential effects on reactive and proactive inhibition and conflict-induced slowing in Parkinson's disease. Exp Brain Res. 2013;226:451–462. 23525560 10.1007/s00221-013-3457-9

[129] Greenhouse I , Gould S, Houser M, Hicks G, Gross J, Aron AR. Stimulation at dorsal and ventral electrode contacts targeted at the subthalamic nucleus has different effects on motor and emotion functions in Parkinson's disease. Neuropsychologia. 2011;49:528–534. 21184765 10.1016/j.neuropsychologia.2010.12.030

[130] Ray NJ , Jenkinson N, Brittain J, et al The role of the subthalamic nucleus in response inhibition: Evidence from deep brain stimulation for Parkinson's disease. Neuropsychologia. 2009;47:2828–2834. 19540864 10.1016/j.neuropsychologia.2009.06.011

[131] Fife KH , Gutierrez-Reed NA, Zell V, et al Causal role for the subthalamic nucleus in interrupting behavior. Elife. 2017;6:e27689. 28742497 10.7554/eLife.27689 PMC5526663

[132] Li B , Nguyen TP, Ma C, Dan Y. Inhibition of impulsive action by projection-defined prefrontal pyramidal neurons. Proc Natl Acad Sci U S A. 2020;117:17278–17287. 32631999 10.1073/pnas.2000523117 PMC7382266

[133] Hershey T , Campbell MC, Videen TO, et al Mapping Go-No-Go performance within the subthalamic nucleus region. Brain. 2010;133(Pt 12):3625–3634. 20855421 10.1093/brain/awq256 PMC2995882

[134] van Wouwe NC , Neimat JS, van den Wildenberg WPM, et al Subthalamic nucleus subregion stimulation modulates inhibitory control. Cereb Cortex Commun. 2020;1:tgaa083. 33381760 10.1093/texcom/tgaa083 PMC7750129

[135] Waldthaler J , Sperlich A, Stüssel C, Steidel K, Timmermann L, Pedrosa DJ. Stimulation of non-motor subthalamic nucleus impairs selective response inhibition via prefrontal connectivity. Brain Commun. 2023;5:fcad121. 37113315 10.1093/braincomms/fcad121 PMC10128876

[136] Manza P , Amandola M, Tatineni V, Li CR, Leung HC. Response inhibition in Parkinson's disease: A meta-analysis of dopaminergic medication and disease duration effects. NPJ Parkinsons Dis. 2017;3:23. 28702504 10.1038/s41531-017-0024-2 PMC5501877

[137] Little S , Pogosyan A, Neal S, et al Adaptive deep brain stimulation in advanced Parkinson disease. Ann Neurol. 2013;74:449–457. 23852650 10.1002/ana.23951 PMC3886292

[138] Little S , Tripoliti E, Beudel M, et al Adaptive deep brain stimulation for Parkinson's disease demonstrates reduced speech side effects compared to conventional stimulation in the acute setting. J Neurol Neurosurg Psychiatry. 2016;87:1388–1389. 27530809 10.1136/jnnp-2016-313518 PMC5136720

[139] Moolchand P , Jones SR, Frank MJ. Biophysical and architectural mechanisms of subthalamic theta under response conflict. J Neurosci. 2022;42:4470–4487. 35477903 10.1523/JNEUROSCI.2433-19.2022 PMC9172290

[140] Ratcliff R , Frank MJ. Reinforcement-based decision making in corticostriatal circuits: Mutual constraints by neurocomputational and diffusion models. Neural Comput. 2012;24:1186–1229. 22295983 10.1162/NECO_a_00270

[141] Spieser L , Servant M, Hasbroucq T, Burle B. Beyond decision! Motor contribution to speed-accuracy trade-off in decision-making. Psychon Bull Rev. 2017;24:950–956. 27757924 10.3758/s13423-016-1172-9

[142] Weindel G , Anders R, Alario FX, Burle B. Assessing model-based inferences in decision making with single-trial response time decomposition. J Exp Psychol Gen. 2021;150:1528–1555. 33764107 10.1037/xge0001010

[143] Bolkan SS , Stone IR, Pinto L, et al Opponent control of behavior by dorsomedial striatal pathways depends on task demands and internal state. Nat Neurosci. 2022;25:345–357. 35260863 10.1038/s41593-022-01021-9 PMC8915388

[144] Derosiere G , Thura D, Cisek P, Duque J. Hasty sensorimotor decisions rely on an overlap of broad and selective changes in motor activity. PLoS Biol. 2022;20:e3001598. 35389982 10.1371/journal.pbio.3001598 PMC9017893

[145] Kehnemouyi YM , Wilkins KB, Anidi CM, Anderson RW, Afzal MF, Bronte-Stewart HM. Modulation of beta bursts in subthalamic sensorimotor circuits predicts improvement in bradykinesia. Brain. 2021;144:473–486. 33301569 10.1093/brain/awaa394 PMC8240742

[146] Wiest C , Tinkhauser G, Pogosyan A, et al Subthalamic deep brain stimulation induces finely-tuned gamma oscillations in the absence of levodopa. Neurobiol Dis. 2021;152:105287. 33549721 10.1016/j.nbd.2021.105287 PMC7116781

[147] Scherrer S , Smith AH, Gowatsky J, et al Impulsivity and compulsivity after subthalamic deep brain stimulation for Parkinson's disease. Front Behav Neurosci. 2020;14:47. 32390809 10.3389/fnbeh.2020.00047 PMC7191054

[148] Debove I , Paschen S, Amstutz D, et al Management of impulse control and related disorders in Parkinson's disease: An expert consensus. Mov Disord. 2024;39:235–248. 38234035 10.1002/mds.29700

[149] Lhommée E , Wojtecki L, Czernecki V, et al Behavioural outcomes of subthalamic stimulation and medical therapy versus medical therapy alone for Parkinson's disease with early motor complications (EARLYSTIM trial): Secondary analysis of an open-label randomised trial. Lancet Neurol. 2018;17:223–231. 29452685 10.1016/S1474-4422(18)30035-8

[150] Baig F , Kelly MJ, Lawton MA, et al Impulse control disorders in Parkinson disease and RBD: A longitudinal study of severity. Neurology. 2019;93:e675–e687. 31311842 10.1212/WNL.0000000000007942 PMC6715510

[151] Eisinger RS , Ramirez-Zamora A, Carbunaru S, et al Medications, deep brain stimulation, and other factors influencing impulse control disorders in Parkinson's disease. Front Neurol. 2019;10:86. 30863353 10.3389/fneur.2019.00086 PMC6399407

[152] Voon V , Napier TC, Frank MJ, et al Impulse control disorders and levodopa-induced dyskinesias in Parkinson's disease: An update. Lancet Neurol. 2017;16:238–250. 28229895 10.1016/S1474-4422(17)30004-2

[153] Herz DM . Neuroscience: Therapy modulates decision-making in Parkinson's disease. Curr Biol. 2024;34:R148–R150. 38412825 10.1016/j.cub.2024.01.031

[154] Dalley JW , Robbins TW. Fractionating impulsivity: Neuropsychiatric implications. Nat Rev Neurosci. 2017;18:158–171. 10.1038/nrn.2017.8 28209979

[155] Yoo SBM , Hayden BY, Pearson JM. Continuous decisions. Philos Trans R Soc Lond B Biol Sci. 2021;376:20190664. 33423634 10.1098/rstb.2019.0664 PMC7815426

[156] Jaskir A , Frank MJ. On the normative advantages of dopamine and striatal opponency for learning and choice. Elife. 2023;12:e85107. 36946371 10.7554/eLife.85107 PMC10198727

[157] Voon V , Pessiglione M, Brezing C, et al Mechanisms underlying dopamine-mediated reward bias in compulsive behaviors. Neuron. 2010;65:135–142. 20152119 10.1016/j.neuron.2009.12.027 PMC2822730

[158] Williams IA , Wilkinson L, Limousin P, Jahanshahi M. Load-Dependent interference of deep brain stimulation of the subthalamic nucleus with switching from automatic to controlled processing during random number generation in Parkinson's disease. J Parkinsons Dis. 2015;5:321–331. 25720447 10.3233/JPD-140355 PMC5389041

[159] Zaehle T , Wagenbreth C, Voges J, Heinze HJ, Galazky I. Effects of deep brain stimulation of the subthalamic nucleus on perceptual decision making. Neuroscience. 2017;343:140–146. 27956065 10.1016/j.neuroscience.2016.11.044

[160] Cherkasova MV , Corrow JC, Taylor A, et al Dopamine replacement remediates risk aversion in Parkinson's disease in a value-independent manner. Parkinsonism Relat Disord. 2019;66:189–194. 31473085 10.1016/j.parkreldis.2019.08.014

[161] Dagher A , Robbins TW. Personality, addiction, dopamine: Insights from Parkinson's disease. Neuron. 2009;61:502–510. 19249271 10.1016/j.neuron.2009.01.031

[162] Florin E , Müller D, Pfeifer J, Barbe MT, Fink GR, Timmermann L. Subthalamic stimulation modulates self-estimation of patients with Parkinson's disease and induces risk-seeking behaviour. Brain. 2013;136(Pt 11):3271–3281. 24071530 10.1093/brain/awt241

[163] Rogers RD , Wielenberg B, Wojtecki L, Elben S, Campbell-Meiklejohn D, Schnitzler A. Deep brain stimulation of the subthalamic nucleus transiently enhances loss-chasing behaviour in patients with Parkinson's disease. Exp Neurol. 2011;231:181–189. 21726554 10.1016/j.expneurol.2011.06.007

[164] Evens R , Stankevich Y, Dshemuchadse M, et al The impact of Parkinson's disease and subthalamic deep brain stimulation on reward processing. Neuropsychologia. 2015;75:11–19. 25976111 10.1016/j.neuropsychologia.2015.05.005

[165] Seinstra M , Wojtecki L, Storzer L, Schnitzler A, Kalenscher T. No effect of subthalamic deep brain stimulation on intertemporal decision-making in Parkinson patients. eNeuro. 2016;3:ENEURO.0019-16.2016. 10.1523/ENEURO.0019-16.2016 PMC4876489 27257622

[166] Seymour B , Barbe M, Dayan P, Shiner T, Dolan R, Fink GR. Deep brain stimulation of the subthalamic nucleus modulates sensitivity to decision outcome value in Parkinson's disease. Sci Rep. 2016;6:32509. 27624437 10.1038/srep32509 PMC5021944

[167] Torta DM , Vizzari V, Castelli L, et al Impulsivities and Parkinson's disease: Delay aversion is not worsened by deep brain stimulation of the subthalamic nucleus. PLoS One. 2012;7:e43261. 22984415 10.1371/journal.pone.0043261 PMC3439437

[168] Boller JK , Barbe MT, Pauls KA, et al Decision-making under risk is improved by both dopaminergic medication and subthalamic stimulation in Parkinson's disease. Exp Neurol. 2014;254:70–77. 24444545 10.1016/j.expneurol.2014.01.005

[169] Castrioto A , Funkiewiez A, Debu B, et al Iowa gambling task impairment in Parkinson's disease can be normalised by reduction of dopaminergic medication after subthalamic stimulation. J Neurol Neurosurg Psychiatry. 2015;86:186–190. 24860137 10.1136/jnnp-2013-307146

[170] Mazzoni A , Rosa M, Carpaneto J, Romito LM, Priori A, Micera S. Subthalamic neural activity patterns anticipate economic risk decisions in gambling. eNeuro. 2018;5:ENEURO.0366-17.2017. 10.1523/ENEURO.0366-17.2017 PMC5810044 29445770

[171] Napier TC , Corvol JC, Grace AA, et al Linking neuroscience with modern concepts of impulse control disorders in Parkinson's disease. Mov Disord. 2015;30:141–149. 25476402 10.1002/mds.26068 PMC4318759

[172] Rosa M , Fumagalli M, Giannicola G, et al Pathological gambling in Parkinson's disease: Subthalamic oscillations during economics decisions. Mov Disord. 2013;28:1644–1652. 23554027 10.1002/mds.25427

[173] Collins AG , Frank MJ. Cognitive control over learning: Creating, clustering, and generalizing task-set structure. Psychol Rev. 2013;120:190–229. 23356780 10.1037/a0030852 PMC3974273

[174] Fine JM , Hayden BY. The whole prefrontal cortex is premotor cortex. Philos Trans R Soc Lond B Biol Sci. 2022;377:20200524. 34957853 10.1098/rstb.2020.0524 PMC8710885

[175] Cisek P , Kalaska JF. Neural mechanisms for interacting with a world full of action choices. Annu Rev Neurosci. 2010;33:269–298. 20345247 10.1146/annurev.neuro.051508.135409

[176] Peixoto D , Verhein JR, Kiani R, et al Decoding and perturbing decision states in real time. Nature. 2021;591:604–609. 33473215 10.1038/s41586-020-03181-9

[177] Krakauer JW , Ghazanfar AA, Gomez-Marin A, MacIver MA, Poeppel D. Neuroscience needs behavior: Correcting a reductionist bias. Neuron. 2017;93:480–490. 28182904 10.1016/j.neuron.2016.12.041

[178] Guidetti M , Marceglia S, Loh A, et al Clinical perspectives of adaptive deep brain stimulation. Brain Stimul. 2021;14:1238–1247. 34371211 10.1016/j.brs.2021.07.063

[179] Meidahl AC , Tinkhauser G, Herz DM, Cagnan H, Debarros J, Brown P. Adaptive deep brain stimulation for movement disorders: The long road to clinical therapy. Mov Disord. 2017;32:810–819. 28597557 10.1002/mds.27022 PMC5482397

[180] Bouthour W , Mégevand P, Donoghue J, Lüscher C, Birbaumer N, Krack P. Biomarkers for closed-loop deep brain stimulation in Parkinson disease and beyond. Nat Rev Neurol. 2019;15:343–352. 30936569 10.1038/s41582-019-0166-4

[181] Athalye VR , Carmena JM, Costa RM. Neural reinforcement: Re-entering and refining neural dynamics leading to desirable outcomes. Curr Opin Neurobiol. 2020;60:145–154. 31877493 10.1016/j.conb.2019.11.023

[182] Turner RS , Desmurget M. Basal ganglia contributions to motor control: A vigorous tutor. Curr Opin Neurobiol. 2010;20:704–716. 20850966 10.1016/j.conb.2010.08.022 PMC3025075

[183] Shadmehr R , Reppert TR, Summerside EM, Yoon T, Ahmed AA. Movement vigor as a reflection of subjective economic utility. Trends Neurosci. 2019;42:323–336. 30878152 10.1016/j.tins.2019.02.003 PMC6486867

[184] Merk T , Peterson V, Köhler R, Haufe S, Richardson RM, Neumann WJ. Machine learning based brain signal decoding for intelligent adaptive deep brain stimulation. Exp Neurol. 2022;351:113993. 35104499 10.1016/j.expneurol.2022.113993 PMC10521329

[185] Mar-Barrutia L , Real E, Segalás C, Bertolín S, Menchón JM, Alonso P. Deep brain stimulation for obsessive-compulsive disorder: A systematic review of worldwide experience after 20 years. World J Psychiatry. 2021;11:659–680. 34631467 10.5498/wjp.v11.i9.659 PMC8474989

